# Gender and cross-country differences in the determinants of sustainable diet intentions: a multigroup analysis of the UK, China, Sweden, and Brazil

**DOI:** 10.3389/fpsyg.2024.1355969

**Published:** 2024-02-29

**Authors:** Emelie Chard, Cecilia Jakobsson Bergstad, Katharine Steentjes, Wouter Poortinga, Christina Demski

**Affiliations:** ^1^Department of Psychology, University of Gothenburg, Göteborg, Sweden; ^2^Centre for Climate Change and Social Transformations, School of Psychology, Cardiff University, Cardiff, United Kingdom; ^3^School of Psychology, Swansea University, Swansea, United Kingdom; ^4^Welsh School of Architecture, Cardiff University, Cardiff, United Kingdom; ^5^Centre for Climate Change and Social Transformations, Department of Psychology, University of Bath, Bath, United Kingdom

**Keywords:** diet, sustainability, gender, country comparisons, behavioral intentions, structural equation modeling

## Abstract

**Introduction:**

Gender differences have been identified in both the engagement in and the determinants of sustainable diet behaviours. However, as engagement in pro-environmental behaviours varies across countries, the consistency of gender differences could follow similar patterns. Understanding the factors underlying gender and country differences in diet intentions is important for determining how to promote sustainable diets in different populations.

**Methods:**

Using survey data from the UK, China, Sweden and Brazil (*N*=4,569), this paper examines the influence of subjective norms, diet-related identity, perceived status of meat consumption, environmental identity and environmental concern on sustainable diet intentions. Multigroup analysis was used to explore gender and country differences in the influence of these variables, and whether gender differences were consistent across the four countries.

**Results:**

The findings show that there are gender differences in the influence of diet-related identity and perceived status of meat consumption, as well as cross-country differences in all factors except subjective norms. Holding a strong diet-related meat identity negatively affected sustainable diet intentions in all groups. Crucially, however, gender differences are not consistent across countries.

**Discussion:**

These results suggest that individuals’ intentions to engage in sustainable diet behaviours are influenced by nationally unique gender associations.

## 1 Introduction

### 1.1 Background

The food industry is one of the largest producers of greenhouse gasses accounting for roughly 35% of global emissions (Xu et al., 2021), making it one area where drastic change is needed in order to mitigate climate change. Animal products contribute a large portion of this, producing double the emissions of plant-based foods (Xu et al., 2021). Furthermore, with roughly a third of all food wasted, 8–10% of greenhouse emissions are produced for foods that are never consumed (United Nations Environment Programme, 2021). Adopting more sustainable diet behaviors such as following a vegan or vegetarian diet, limiting food waste and eating locally produced food therefore has great potential to reduce carbon emissions associated with our diets (Ivanova et al., 2020). Understanding the determinants of these behaviors should therefore be a first step toward encouraging lower emission diets.

Previous research has identified several factors that influence pro-environmental behaviors. For example, the theory of planned behavior (TPB; Ajzen, 1991) has frequently been applied to several pro-environmental behaviors (Liobikiene et al., 2016; Oztekin et al., 2017; Hu et al., 2021) and suggests that intentions, and subsequent engagement with these behaviors, are predicted by attitudes toward the behavior, perceived behavioral control (whether a person considers the behavior to be under their control) and subjective norms (the perceived social pressure to engage in that behavior). Several researchers have proposed extending this model to capture other factors that may influence these behaviors including environmental concern (Hu et al., 2021) and self-identity, referring to beliefs about the self and who a person believes themselves to be (Sparks and Shepherd, 1992). Whitmarsh and O’Neill (2010) even found self-identity, both specific to the target behavior and of being ‘pro-environmental’, indicating how important these concepts are to an individual’s identity, influenced some pro-environmental behaviors over and above the variables in the TPB.

However, there is an extensive literature showing gender differences in a range of pro-environmental behaviors (Zelezny et al., 2000; Xiao and McCright, 2014), which extend to women tending to show higher engagement in several sustainable diet behaviors (Asher et al., 2014; Love and Sulikowski, 2018; Cantaragiu, 2019). Similarly, there is an extensive literature showing cross-national differences in pro-environmental behaviors (Vicente-Molina et al., 2013; Liobikiene et al., 2016) although few studies have explored the cultural differences in sustainable diet behaviors.

Exploring the determinants of these sustainable diet behaviors across genders and countries should help to understand these differences. This understanding will ultimately help reduce any gender or country gap in who engages with these behaviors, for example, through designing interventions to lower the carbon emissions associated with our diet which target the specific factors that influence different populations. This study therefore aims to examine gender differences in sustainable diet intentions and their determinants in four countries; the UK, Brazil, Sweden and China. These four countries represent national contexts with different dietary cultures, climate ambitions, and rates of development. Because the highest proportion of future emissions is predicted to come from emerging economies (Rong, 2010), these countries were selected in order to compare the UK to two rapidly developing countries (China, Brazil), together with a country (Sweden) with ambitious climate change policies (CCPI, 2018). Brazil represents a country from the Global South, with high vulnerability to climate change impacts and a dietary culture heavily centered around meat consumption (OECD, 2023). China is among the fastest growing economies around the world (Harvard Growth Lab, 2023), with a food culture that values freshness but China was ranked third highest country for pork consumption (Lin, 2000; OECD, 2023). The UK and Sweden represent European countries from the Global North with different approaches to reducing their historic contribution to climate change. Sweden has the lowest greenhouse gas emission *per capita* of all European countries due to its energy being largely generated by renewable energy sources. The UK has recently lowered their net zero ambitions and are scaling back policies such as the ban on petrol and diesel cars or gas boilers (Prime Minister’s Office, 2023). Traditional UK food does often contain meat but meat consumption has gone down in recent years (Tedstone, 2020). Exploring meat reduction behaviors across these different countries and exploring potential gender differences will provide extremely valuable insights into the role of gender in meat consumption and reduction strategies.

Existing evidence exploring engagement with pro-environmental behaviors shows that the influence of several determinants of these behaviors, such as attitudes (Oztekin et al., 2017; Vicente-Molina et al., 2018) and perceived behavioral control (Oztekin et al., 2017) vary between men and women. In relation to diet, there are gender differences in the perceived barriers to adopting more sustainable diet behaviors (Mäkiniemi and Vainio, 2014).

Various scholars have argued that gender differences in pro-environmental behavior stem from differences in the way men and women are socialized to engage with the environment, specifically that women are more likely to engage in pro-environmental behaviors as a result of socialized values such as caretaking, compassion and communality (Bloodhart and Swim, 2020). This is supported by research showing that women have stronger feelings of social responsibility in relation to the environment (Zelezny et al., 2000) and are more likely to cite pro-social motivations for following a vegetarian diet, such as the benefits to animal welfare and the environment (Trocchia and Janda, 2003; Rosenfeld, 2020). In contrast, men typically have more self-enhancing values that are considered incongruent with pro-environmentalism (Dietz et al., 2002). These values are typically associated with more environmentally destructive behaviors such as car ownership and meat-eating (Polk, 2004; Rozin et al., 2012). Particularly in the Western cultures where most of this research has been conducted, meat consumption is perceived as masculine (Timeo and Suitner, 2018), whereas more healthy and sustainable diets are seen as more feminine (Thomas, 2016; Modlinska et al., 2020; Rodrigues et al., 2020). These gender associations are persistent with Graziani et al.’s (2021) finding that even pre-school age children associated meat with men and vegetables with women. These stereotypes may then contribute to the existence of gender differences in diet (Rodrigues et al., 2020; Graziani et al., 2021) with individuals more likely to consume foods that match their gender identity (Timeo and Suitner, 2018; Ekebas-Turedi et al., 2021). Gal and Wilkie (2010) even found that men are likely to forgo their personal preferences to eat foods which match their masculine identity. Men who follow a more sustainable diet still differ from women in their reasonings for doing so, by avoiding feminine stereotypes and instead aligning their diet with traditionally masculine values such as courage and self-control (Greenebaum and Dexter, 2018; Mycek, 2018). Furthermore, men and women may experience different social pressures regarding sustainable diet behaviors, likely due to these gendered stereotypes. Men who follow a vegetarian diet expect less support and often face more prejudice than women who follow a vegetarian diet (Worsley and Skrzypiec, 1997; Modlinska et al., 2020), including having their masculinity questioned (Torti, 2017). It is therefore likely that social norms, theorized as one of the key determinants of behavior in the TPB (Ajzen, 1991) play a different role for men and women.

While previous research has also found differences in engagement with pro-environmental behavior between countries (Vicente-Molina et al., 2013; Liobikiene et al., 2016), research into the determinants of these behaviors has been criticized for largely overlooking the role of culture (Popovic et al., 2019). Some studies have found that the levels of several of these common determinants vary across countries according to several cultural variables, including collectivism vs. individualism (Husted, 2005; Cho et al., 2013; Halder et al., 2020), affluence (Diekmann and Franzen, 1999; Duroy, 2008; Aral and López-Sintas, 2021) and education (Knight, 2016; Aral and López-Sintas, 2021). The strength of influence these determinants have on pro-environmental behaviors has also been found to depend on several country-level variables. For example, within EU countries, country affluence has been found to moderate the influence of subjective norms (Liobikiene et al., 2016; Aral and López-Sintas, 2021) and attitudes (Aral and López-Sintas, 2021) on various pro-environmental behaviors. It is likely that more affluent, developed countries have an improved ability to focus on environmental issues once basic needs are met and resources are less scarce (Inglehart, 1995; Diekmann and Franzen, 1999). This may explain why countries with a higher GDP *per capita*, including both the UK and Sweden, tend to have better environmental performance (Environmental Performance Index, 2022). This, as a result, may create different cultural norms and attitudes concerning these behaviors. Likewise, in relation to diet, the influence of these behavioral determinants may depend on the specific food culture of that country, referring to the shared eating practices of people with the same cultural identity (Munz Fernandes et al., 2021; Nguyen and Platow, 2021). Meat-eating is typically an important, yet contentious, part of this, demonstrated by how widely attitudes toward both beef and vegetarians varies between countries (Ruby et al., 2016). In particular, in Brazil where the food culture includes a high beef consumption (Travassos et al., 2020), attitudes toward beef are mostly positive (Ruby et al., 2016). In contrast, some food cultures already align with more sustainable diet practices, such as the predominantly plant-based Mediterranean diet (Zamora-Ros et al., 2013; Serra-Majem and Medina, 2015), which may result in stronger attitudes and norms in favor of sustainable diets behaviors. Wolstenholme et al. (2021) suggested that the higher prevalence of the Mediterranean diet in Italy could explain why perceived behavioral control only influenced intentions to reduce meat consumption in UK participants and not participants from Italy, as the food culture in UK is more centered around meat and so people may perceive there to be less sustainable alternatives available.

Cultural and gender differences may also intersect. While the gender gap in pro-environmental behaviors has been identified in multiple countries (Zelezny et al., 2000; Vicente-Molina et al., 2013), there is little research into how consistent these gender differences are (Liobikiene et al., 2016; Oztekin et al., 2017). Gender differences may be expected to vary between countries as gendered roles and beliefs are partly a product of the social environment and thus are flexible to change between countries (Eagly and Wood, 1999; Zafra and Garcia-Retamero, 2011). For example, adherence to traditional masculinity, a concept that has been theorized to explain gender differences in meat-eating (Timeo and Suitner, 2018), has been found to vary between countries with American men endorsing more traditionally masculine norms than Italian men (Tager and Good, 2005). Likewise, the gendered associations of certain foods are not consistent across cultures (Rodrigues et al., 2020; Ekebas-Turedi et al., 2021) with, for example, high fat foods being perceived as masculine in the US but feminine in Turkey. This suggests that gender differences in sustainable diet behaviors may vary according to how these foods are viewed within each country.

### 1.2 Aims of the study

Overall, current evidence indicates that the importance of common determinants of pro-environmental behaviors varies across both gender (Oztekin et al., 2017; Vicente-Molina et al., 2018) and countries (Vicente-Molina et al., 2013; Liobikiene et al., 2016). However, there is limited research on sustainable diet behaviors. Furthermore, it is not clear whether potential gender differences in the determinants of sustainable diet behaviors are consistent across countries. This is important as it is unclear whether the same mechanisms are responsible for producing a gender gap in sustainable diet behaviors across countries and thus whether the same interventions could be used to address this gap across countries. This study therefore aims to explore gender and country differences in the influence of subjective norms, diet-related identity, perceived status of meat consumption, environmental identity and environmental concern on sustainable diet intentions in four countries (i.e., the UK, China, Sweden and Brazil). These countries were chosen as they represent a diverse range of cultural contexts, varying in levels of development, environmental performance and the degree to which the culture is individualistic or collectivistic. Furthermore, this sample expands beyond Western countries where most of this research has typically been focused. Crucially, this study then aims to explore whether gender differences are consistent across these four countries.

To explore how determinants of sustainable diet intentions varies across gender and country, this study will use multigroup analysis. Multigroup analysis is a technique used to explore the moderating role of group variables by comparing how one model applies to different groups. Several studies have previously applied this method to identify how the determinants of other pro-environmental behaviors, including car sharing and green buying intentions, vary across a number of sociodemographic groups, such as gender, age, education and income (Bautista, 2019; Kaur et al., 2022; Li and Zhang, 2023). This is a valuable technique for environmental psychology as it provides detailed insights into group differences which can aid in tailoring environmental communication and interventions to encourage pro-environmental behavior across groups (Alomari et al., 2022).

## 2 Method

### 2.1 Participants and procedure

This study used survey data collected by the Centre of Climate Change and Social Transformations (CAST). The survey was developed through an international collaboration between researchers from the UK, China, Sweden, and Brazil and was administered in these countries. These countries were selected in order to compare the UK context with countries with different emissions trajectories (Raupach et al., 2007) and cultural characteristics (Pettifor et al., 2017). For example, China and Brazil represent two rapidly developing countries, while Sweden represents a country with ambitious climate change policies (CCPI, 2018). The online survey was conducted between 29th September and 26th October 2020 and took around 20 min to complete. The total number of survey responses was *N* = 4,724. As the focus of the current paper is on gender, individuals who did not identify as male or female were excluded from the analyses (describing gender in a different way, *N* = 14; prefer not to say, *N* = 8; missing data, *N* = 133). This left a sample of 4,569 participants [N_female_ = 2,303; M_age_(SD) = 46.0 (15.7)] from the UK (*N* = 1,842, N_female_ = 963), China (*N* = 807, N_female_ = 363), Sweden (*N* = 963, N_female_ = 479), and Brazil (*N* = 957, N_female_ = 498). Quotas for gender, age, region, and socioeconomic status were used to ensure representative samples. Further demographic information is reported in the Supplementary material. Respondents were recruited through external panels and received compensation for participating in the form of either credit, payment or entry into prize draws. The procedure and analysis plan for this study were pre-registered on the Open Science Framework (osf.io/pxnqc)^1^ and used data collected for a larger research project. The survey received ethical approval from Cardiff University, School of Psychology Research Ethics Committee, project number EC.20.08.11.6068. Respondents provided informed consent by electronically clicking a consent statement before moving on to the online questionnaire.

### 2.2 Measures

The survey covered questions about the four areas of diet, transport, home heating and material consumption, as well as measures of individual characteristics and demographic variables. The complete survey consisted of *ca.* 65 questions including matrix questions with multiple statements and 3 to 5 open ended questions. In this paper we only used questions about diet, in particular questions on sustainable diet intentions, social norms, perceived behavioral control, diet-related identity, perceived status of meat consumption, environmental identity, and environmental concern. The survey items used to measure these variables are shown in Table 1.

**Table 1:** Survey items proposed to measure sustainable diet intentions, social norms, perceived behavioral control, diet-related identity, perceived status of meat consumption, environmental identity, and environmental concern.

Question		Response scale
Sustainable diet intentions		
*Please indicate how likely or unlikely you are to take each of the following actions in the next 12 months?*		1 (very unlikely) to 5 (very likely)
Q055_1	Eat fewer calories a day to reduce consumption	
Q055_2	Plan meals ahead to avoid food waste	
Q055_3	Follow a vegan diet	
Q055_4	Follow a vegetarian diet	
Q055_5	Buy locally produced food	
Social norms		
*To what extent do you agree or disagree with the following statements?*		1 (strongly disagree) to 5 (strongly agree)
Q021_7	Most of my friends follow a vegetarian (meat free) diet	
Q021_8	Most people close to me eat meat every day^1^	
Q021_9	I feel that most people close to me would disapprove if I stopped eating meat^1^	
Q021_10	Being vegetarian is frowned upon by my family and friends^1^	
Perceived behavioral control		
*To what extent do you agree or disagree with the following statements?*		1 (strongly disagree) to 5 (strongly agree)
Q021_4	I feel in control of making decisions about what food I eat	
Q021_5	There are a lot of vegetarian or meat-free options to choose from (e.g., in supermarkets/restaurants)	
Diet-related identity		
*To what extent do you agree or disagree with the following statements?*		1 (strongly disagree) to 5 (strongly agree)
Q058_1	I am not the type of person to become vegetarian	
Q058_2	Eating meat is an important part of who I am	
Perceived status of meat consumption		
*To what extent do you agree or disagree with the following statements?*		1 (strongly disagree) to 5 (strongly agree)
Q059_1	Eating meat is associated with high status in my society	
Q059_2	If you are vegetarian or vegan, others might view you negatively	
Environmental identity		
*To what extent do you agree or disagree with the following statements?*		1 (strongly disagree) to 5 (strongly agree)
Q062_1	Being environmentally friendly is an important part of who I am	
Q062_2	I think of myself as someone who is very concerned with environmental issues	
Environmental concern		
Q07	How worried, if at all, are you about climate change?	1 (not at all worried) to 5 (extremely worried)
*How serious a threat, if at all, is climate change to each of the following?*		1 (not at all serious) to 5 (extremely serious)
Q010_1	You and your family	
Q010_2	[COUNTRY] as a whole^2^	
Q010_3	People in less developed countries	

^1^Scores for these questions were reversed. ^2^[COUNTRY] referred to the UK, China, Sweden and Brazil in the respective countries.

Sustainable diet intentions was the sole dependent variable and measured participants willingness to engage in different sustainable diet behaviors. Social norms included two items measuring descriptive social norms (Q021_7 and Q021_8), how common a person perceives a behavior to be, and two items measuring injunctive social norms (Q021_9 and Q021_10), the expected rewards, or approval, of a behavior (Cialdini et al., 1990; Thøgersen, 2006). Injunctive social norms will here be referred to as subjective norms, in line with the terminology of the TPB (Ajzen, 1991). Perceived behavioral control related to the level of control a person believes they have over engaging in sustainable diet behaviors. Diet-related identity refers to how central a person’s eating behaviors, specifically meat-eating, are to their self-identity. Perceived status of meat consumption measured the perceived level of approval or status associated with certain diets within society. Environmental identity refers to how central a person’s concern for the environment is to their self-identity. Environmental concern measured the level of concern a person feels about the threat of climate change.

### 2.3 Data analysis

Structural equation models (SEM) were constructed using AMOS version 28, using maximum likelihood estimation methods, with data stored in SPSS version 28. The data were screened for exclusions, missing values, and normality. Little’s (1988) test of data missing completely at random (MCAR) and multiple imputation analysis were used to identify any patterns in the missing data. Missing values were then imputed using the regression method in AMOS. To assess normality, univariate kurtosis for each item below 7 (West et al., 1995) and multivariate kurtosis below 5 (Byrne, 2016) were taken to indicate normally distributed data. Bootstrapping with 1,000 samples was run to account for departures from normality and to allow for the calculation of standard errors and confidence intervals.

**Figure 1:**
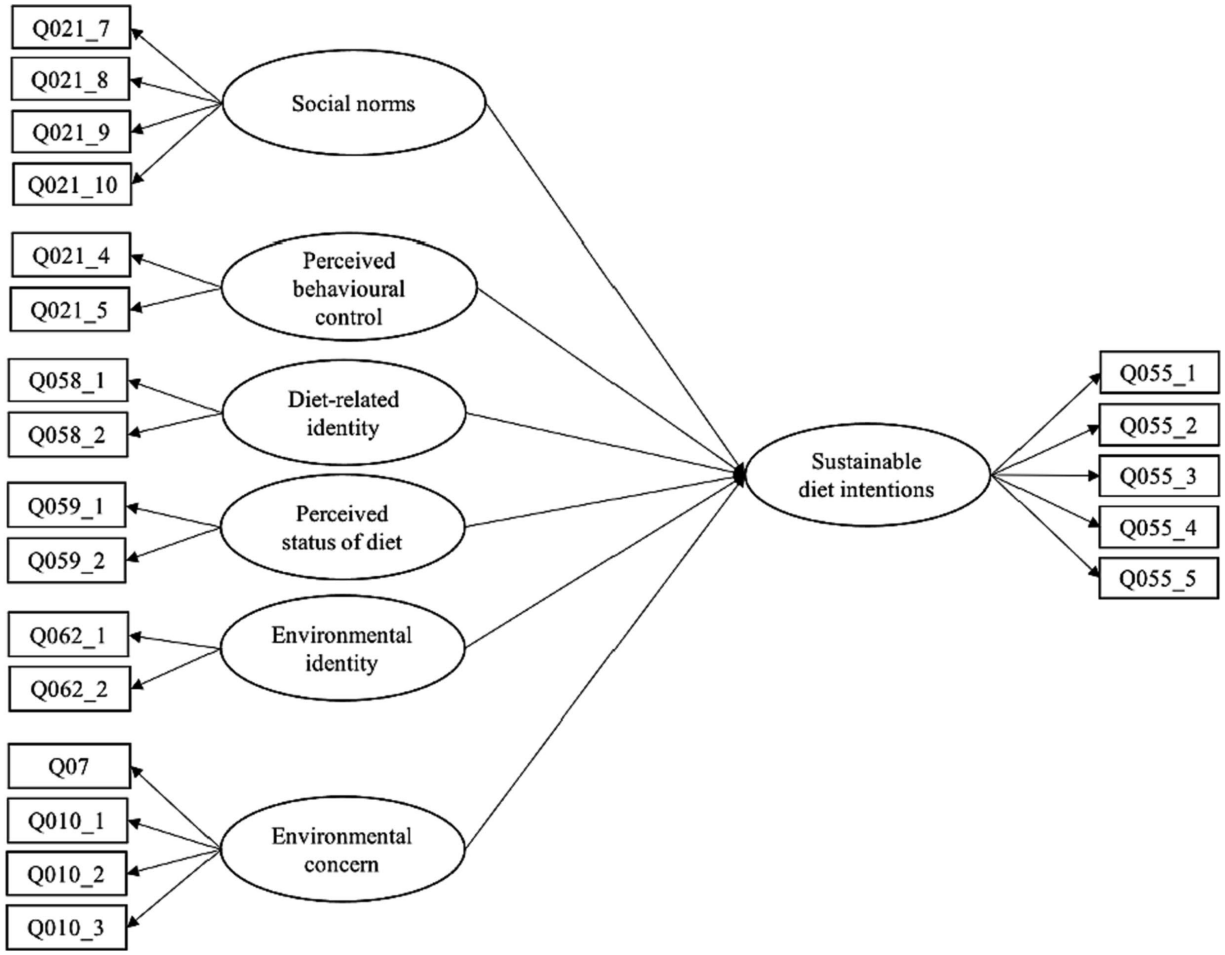
Initial proposed structural equation model of factors influencing sustainable diet intentions.

First, a confirmatory factor analysis (CFA) was conducted on the initial proposed model shown in Figure 1, using the phantom variable approach (Crowson, 2021). Factor loadings and composite reliability were inspected, and the model adjusted based on the results of the CFA (see *Results* section).

Second, a multigroup analysis was conducted to examine gender differences in the determinants of sustainable diet intentions. Model fit indices of an unconstrained model and a model with structural weights constrained to be equal across genders were compared to determine whether the model was variant across gender. A non-significant chi-square (χ^2^) value (at the 0.05 level), comparative fit index (CFI) of 0.95 or higher (Shi et al., 2019), root mean square error of approximation (RMSEA) of 0.06 or less (Shi et al., 2019) and standardized root mean squared residual (SRMR) of 0.05 or less (Schumacker and Lomax, 2016) were considered to indicate good model fit. Each parameter was then constrained individually to determine which factors are variant in sustainable diet intentions across men and women.

Third, similar multigroup analyses were conducted to test whether the determinants of sustainable diet intentions are variant across the UK, China, Sweden and Brazil. Similar to the multigroup analysis for gender, each parameter was then constrained individually to identify which factors are variant in sustainable diet intentions across the four countries.

Fourth, and finally, a series of multigroup analyses was run with the sample divided into eight gender x country subgroups, each representing a subset of the sample (UK men, UK women, etc.). The two gender groups of each country were then compared to explore gender differences within each country.

## 3 Results

### 3.1 Data screening

An initial screening of the data revealed that there were a small number of missing values within the data, with up to 4.3% missing in one variable. Little’s (1988) Missing Completely at Random (MCAR) test for Multivariate Data was significant [χ^2^(1871) = 2278.77, *p* < 0.001] indicating that the missing data cannot be assumed to be completely at random. However, as this test is sensitive to sample size, a sample this large is likely to produce a significant result. Further analysis through the multiple imputation function in SPSS revealed that 86.9% of cases and 98.8% of values were complete. In addition, there were no distinct patterns in the missing data meaning that the data could be considered missing at random (MAR). Screening for normality revealed that the data were partially normally distributed as the univariate kurtosis for each item is below 7 (West et al., 1995). However, the critical ratio of the multivariate kurtosis was much greater than 5, at 89.00, indicating that the data may not be normally distributed (Byrne, 2016). Bootstrapping with 1,000 samples was therefore used in subsequent analyses.

### 3.2 Confirmatory factor analysis

A CFA was run to test that the items loaded onto their intended latent variables. This initial proposed model (Figure 1) showed poor model fit [χ^2^(169) = 5655.07, *p* < 0.001, CFI = 0.847, RMSEA = 0.084, SRMR = 0.093]. Factor loadings and composite reliability, with 95% confidence intervals, were then calculated for each variable to inform re-specifications to the model. For factors with more than two items, items with factor loadings below the cut-off value of 0.4 (Stevens, 1992) were considered for exclusion. Composite reliability was calculated again for the respecified model and if the confidence intervals excluded the estimates from the previous model, the items were deleted. Out of the four items used to measure social norms, the factor loadings of two items fell below 0.4. Composite reliability for this variable was estimated at 0.54 (95% CI [0.52, 0.57]). However, as this variable was intended to measure both descriptive and subjective norms, and the items to be included/excluded were divided by these constructs, the variable was split into two separate variables to represent the two types of social norms. These new specifications to the model resulted in high factor loadings for subjective norms and moderate factor loadings for the descriptive norms. Composite reliability was calculated to be 0.73 (95% CI [0.71, 0.75]) for subjective norms and 0.39 (95% CI [0.35, 0.43]) for descriptive norms. The reliability for descriptive norms is well below even more lenient thresholds (Hair et al., 2014). This factor was therefore excluded from further analyses.

Both items for perceived behavioral control showed low factor loadings, with one item at 0.38 and the other only at 0.44. In addition, composite reliability for this variable was very low 0.28 (95% CI [0.24, 0.33]). It was concluded that this variable could not be reliably measured and therefore was removed from the model.

The items used to measure diet-related identity, perceived status of meat consumption and environmental identity all had sufficiently high factor loadings on their latent variables (between 0.62 and 0.92). Composite reliability for these factors were calculated as 0.77 (95% CI [0.75, 0.78]) for diet-related identity, 0.57 (95% CI [0.54, 0.60]) for perceived status of meat consumption and 0.87 (95% CI [0.86, 0.88]) for environmental identity.

All items for environmental concern had high factor loadings ranging from 0.73 to 0.92. This variable also showed high composite reliability at 0.88 (95% CI [0.88, 0.89]), meaning no exclusions were made to this variable.

Factor loadings of two of the sustainable diet intention items fell below the cut-off value of 0.4 (Stevens, 1992). The composite reliability estimate of this variable was 0.73 (95% CI [0.71, 0.74]). After these two items were excluded, two items showed high factor loadings to the updated variable, but one item was only just above cut-off threshold (at 0.405). In addition, composite reliability for this variable increased to 0.80 (95% CI [0.78, 0.81]), excluding the confidence interval estimates from the original variable. However, as this variable covers a broad range of behaviors, further exclusions were considered to create a more targeted variable focused on just intentions to follow a vegetarian or vegan diet. These exclusions were considered if the composite reliability for the updated variable fell below the recommended threshold for good reliability of 0.8 (Netemeyer et al., 2003; Catalán, 2019). As the confidence interval fell below this threshold, factor loadings and composite reliability for the targeted variable were calculated. Factor loadings for this item were both high and composite reliability was found to be 0.87 (95% CI [0.86, 0.88]). As the confidence intervals for this variable exclude the estimates for the previous model, the targeted intentions variable was included in the model.

**Figure 2:**
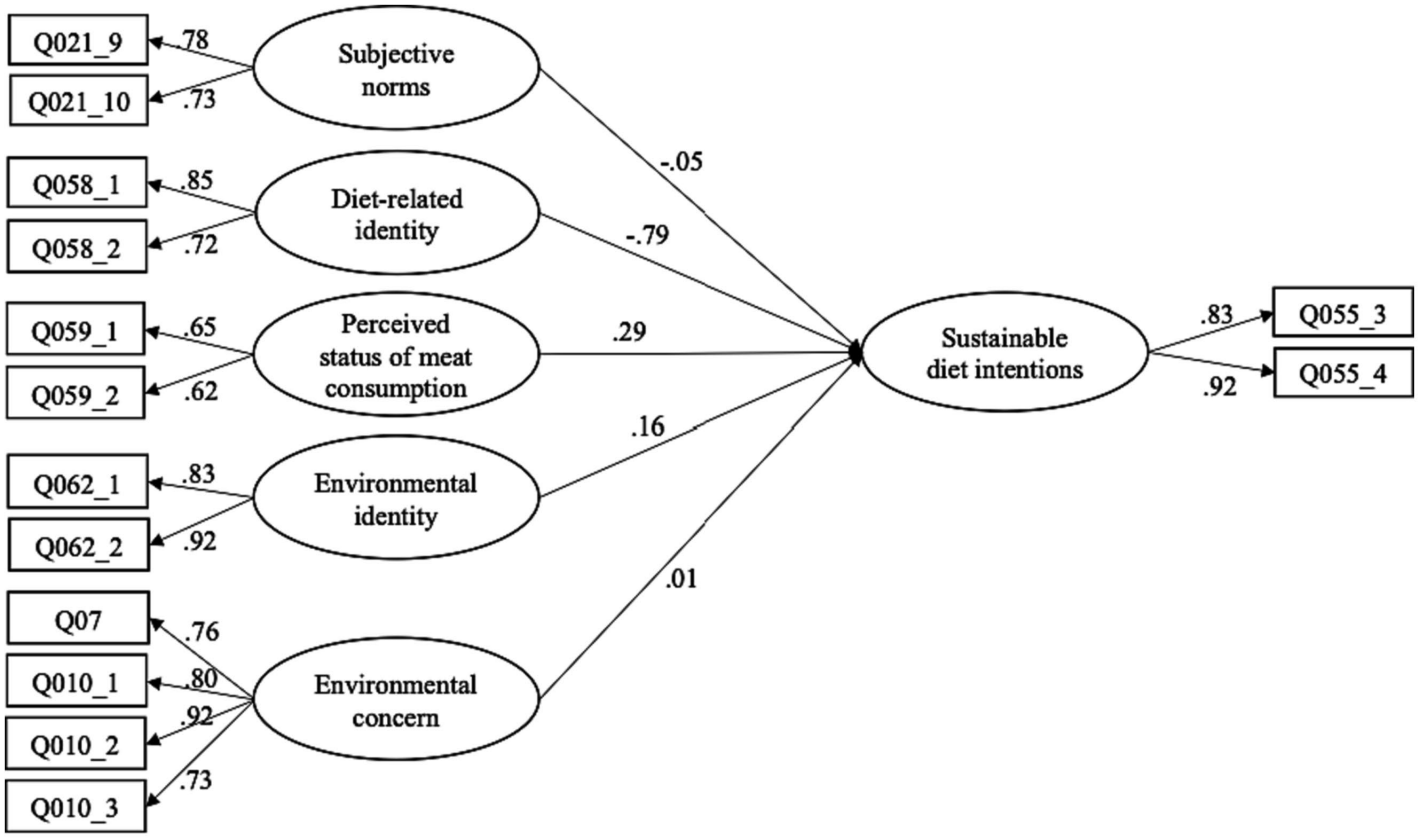
Final structural equation model of factors influencing sustainable diet intentions with standardized path coefficients.

The final model (Figure 2) showed good model fit after these adjustments [χ^2^(62) = 1289.98, *p* < 0.001, CFI = 0.959, RMSEA = 0.066, SRMR = 0.038], with composite reliability estimates ranging from 0.57 to 0.88. This indicated that analysis could proceed with the assumption that these items measure their intended latent variables. Correlations between these variables and descriptive statistics for each of these variables across gender, country and gender x country groups are reported in the Supplementary material. The Supplementary material also includes a two-way ANOVA to compare the differences in these variables across gender and country, as well as the interaction between gender and country.

### 3.3 Gender differences in the factors influencing sustainable diet intentions

A multigroup analysis was run to explore gender differences in the influence of these factors influencing sustainable diet intentions. Model fit indices were good and similar for both the unconstrained [χ^2^(124) = 1353.86, *p* < 0.001, CFI = 0.958, RMSEA = 0.047, SRMR = 0.040] and constrained model [χ^2^(151) = 1662.03, *p* < 0.001, CFI = 0.948, RMSEA = 0.047, SRMR = 0.040]. The difference between the two models was however significant [χ^2^(27) = 308.18, *p* < 0.001], indicating that the model is variant across genders. Further analyses show that constraints on the pathways of subjective norms [χ^2^(1) = 0.94, *p* = 0.332], environmental identity [χ^2^(1) = 0.42, *p* = 0.516] and environmental concern [χ^2^(1) = 0.002^2^, *p* = 0.962] did not result in significant differences from the unconstrained model. Constraining the pathways from diet-related identity [χ^2^(1) = 14.64, *p* < 0.001] and perceived status of meat consumption [χ^2^(1) = 10.84, *p* = 0.001] did however change the model fit, suggesting that the influence of these factors is variant across gender.

Path coefficients for each of these relationships in men and women are shown in Table 2. Only diet-related identity, perceived status of meat consumption and environmental identity significantly influenced sustainable diet behaviors in both genders. Perceived status of meat consumption was found to be more influential in men than in women. Although the multigroup analysis found diet-related identity to be variant across genders, the factor appeared important in both genders, although slightly more so in men. Therefore, only moderate support is found for the existence of gender differences in the factors that influence sustainable diet intentions.

**Table 2:** Total effects of subjective norms, diet-related identity, perceived status of meat consumption, environmental identity and environmental concern on sustainable diet intentions for men and women.

	B (SE)	ß	95% CI
Subjective norms**→**Sustainable diet intentions			
Men	−0.015 (0.055)	−0.016	−0.104, 0.088
Women	−0.078 (0.045)	−0.072	−0.151, 0.006
Diet-related identity**→**Sustainable diet intentions			
Men	−0.880 (0.050)	−0.817*	−0.875, −0.750
Women	−0.692 (0.028)	−0.779*	−0.823, −0.737
Perceived status of meat consumption**→**Sustainable diet intentions			
Men	0.604 (0.087)	0.416*	0.302, 0.525
Women	0.307 (0.059)	0.202*	0.120, 0.275
Environmental identity**→**Sustainable diet intentions			
Men	0.188 (0.034)	0.152*	0.098, 0.206
Women	0.218 (0.033)	0.168*	0.119, 0.217
Environmental concern**→**Sustainable diet intentions			
Men	0.004 (0.036)	0.003	−0.054, 0.057
Women	0.006 (0.028)	0.005	−0.040, 0.046

*p < 0.05. Results based off analyses with 1,000 bootstrap samples.

### 3.4 Country differences in factors influencing sustainable diet intentions

Another multigroup analysis was run with the sample divided by country. Model fit indices were much better for the unconstrained model [χ^2^(248) = 1681.49, *p* < 0.001, CFI = 0.951, RMSEA = 0.036, SRMR = 0.041] than for the constrained model [χ^2^(329) = 4773.57, *p* < 0.001, CFI = 0.849, RMSEA = 0.054, SRMR = 0.048]. The difference between the two models was significant [χ^2^(81) = 3092.08, *p* < 0.001], indicating that the model is variant across countries. Individual analysis of the parameters showed that constraints on the pathway of subjective norms did not result in a significant difference from the unconstrained model [χ^2^(3) = 6.26, *p* = 0.100]. Constraining the pathways of diet-related identity [χ^2^(3) = 19.19, *p* < 0.001], perceived status of meat consumption [χ^2^(3) = 48.69, *p* < 0.001], environmental identity [χ^2^(3) = 26.98, *p* < 0.001] and environmental concern [χ^2^(3) = 13.09, *p* = 0.004] did however change the model fit, suggesting that the influence of these factors is variant across the four countries.

Table 3 shows the total effects of these variables on sustainable diet intentions across countries. It suggests that diet-related identity is the only factor to strongly influence sustainable diet intentions in all four countries. Perceived status of meat consumption influences sustainable diet intentions in all countries but Brazil, and environmental identity influences sustainable diet intentions in all countries but the UK. Environmental concern was only found to influence sustainable diet intentions in the UK and Brazil. Subjective norms were not found to influence intentions in any country. Overall, these results show support for country differences in the factors that influence sustainable diet behaviors.

**Table 3:** Total effects of subjective norms, diet-related identity, perceived status of meat consumption, environmental identity and environmental concern on sustainable diet intentions for respondents from the UK, China, Sweden, and Brazil.

	B (SE)	ß	95% CI
Subjective norms**→** Sustainable diet intentions			
UK	−0.105 (0.057)	−0.103	−0.204, 0.011
China	0.073 (0.172)	0.060	−0.172, 0.295
Sweden	0.187 (0.221)	0.108	−0.069, 0.437
Brazil	−0.113 (0.080)	−0.107	−0.253, 0.048
Diet-related identity**→** Sustainable diet intentions			
UK	−0.632 (0.029)	−0.832*	−0.878, −0.782
China	−1.013 (0.145)	−0.795*	−0.998, −0.626
Sweden	−0.784 (0.060)	−0.850*	−0.945, −0.769
Brazil	−0.801 (0.074)	−0.722*	−0.799, −0.642
Perceived status of meat consumption**→** Sustainable diet intentions			
UK	0.304 (0.075)	0.245*	0.133, 0.369
China	0.996 (0.118)	0.696*	0.558, 0.843
Sweden	0.439 (0.322)	0.222*	0.029, 0.564
Brazil	0.109 (130)	0.071	−0.080, 0.266
Environmental identity**→** Sustainable diet intentions			
UK	0.039 (0.029)	0.039	−0.019, 0.094
China	0.356 (0.096)	0.171*	0.073, 0.258
Sweden	0.113 (0.055)	0.096*	0.012, 0.185
Brazil	0.289 (0.054)	0.222*	0.141, 0.298
Environmental concern**→** Sustainable diet intentions			
UK	0.105 (0.029)	0.104*	0.045, 0.155
China	0.059 (0.074)	0.031	−0.052, 0.111
Sweden	0.019 (0.057)	0.016	−0.073, 0.094
Brazil	−0.139 (0.064)	−0.071*	−0.135, −0.008

*p < 0.05. Results based off analyses with 1,000 bootstrap samples.

### 3.5 Gender differences in the factors influencing sustainable diet intentions in the UK, China, Sweden, and Brazil

A final series of multigroup analyses was run with the eight gender x country subgroups of the sample. Model fit indices showed much better fit for the unconstrained model [χ^2^(496) = 2009.17, *p* < 0.001, CFI = 0.948, RMSEA = 0.026, SRMR = 0.046] than for the constrained model [χ^2^(685) = 5488.70, *p* < 0.001, CFI = 0.834, RMSEA = 0.039, SRMR = 0.072]. The difference between the two models was significant [χ^2^(189) = 3479.53, *p* < 0.001], suggesting that the model is variant across the eight gender x country groups. Further analyses showed that constraints on the pathways from subjective norms [χ^2^(7) = 19.05, *p* = 0.008], diet-related identity [χ^2^(7) = 29.19, *p* < 0.001], perceived status of meat consumption [χ^2^(7) = 65.71, *p* < 0.001] and environmental identity [χ^2^(7) = 28.94, *p* < 0.001] to sustainable diet intentions resulted in a worse model fit, indicating that the influence of these variables does differ between the groups. The influence of environmental concern [χ^2^(7) = 12.53, *p* = 0.085] was not found to vary across the groups.

To explore gender differences within each country, the parameters for men and women in each country were constrained to be equal and compared to the unconstrained model. In the UK, there were only gender differences in environmental identity [χ^2^(1) = 4.34, *p* = 0.037]. The influence of subjective norms [χ^2^(1) = 0.04, *p* = 0.837], diet-related identity [χ^2^(1) = 1.86, *p* = 0.172], perceived status of meat consumption [χ^2^(1) = 0.06, *p* = 0.815] and environmental concern [χ^2^(1) = 1.42, *p* = 0.234] did not vary across genders. In China, there were no gender differences in subjective norms [χ^2^(1) = 0.75, *p* = 0.387], diet-related identity [χ^2^(1) = 1.47, *p* = 0.225], perceived status of meat consumption [χ^2^(1) = 0.20, *p* = 0.652], environmental identity [χ^2^(1) = 0.282, *p* = 0.595] or environmental concern [χ^2^(1) = 0.085, *p* = 0.771]. In Sweden, gender differences were found in both subjective norms [χ^2^(1) = 5.26, *p* = 0.022] and perceived status of meat consumption [χ^2^(1) = 5.97, *p* = 0.015]. The influence of diet-related identity [χ^2^(1) = 3.01, *p* = 0.083], environmental identity [χ^2^(1) = 1.48, *p* = 0.224] and environmental concern [χ^2^(1) = 0.05, *p* = 0.826] did not vary across genders. In Brazil, gender differences were found in subjective norms [χ^2^(1) = 4.33, *p* = 0.037], diet-related identity [χ^2^(1) = 4.00, *p* = 0.046] and perceived status of meat consumption [χ^2^(1) = 15.09, *p* < 0.001]. The influence of environmental identity [χ^2^(1) = 0.043, *p* = 0.835] and environmental concern [χ^2^(1) = 0.626, *p* = 0.429] did not vary across genders.

The importance of the factors in each subgroup is shown in Table 4. The results suggest that subjective norms are only an important factor for Swedish men and Brazilian women. However, the direction of this influence varies between the groups, with Brazilian women appearing more reactive to and defiant of subjective norms. Diet-related identity was a significant factor in all groups, with a consistent negative association with sustainable diet intentions. Perceived status of meat consumption was found to be an important factor in all groups but Swedish and Brazilian women, with a positive association with sustainable diet intentions in all other groups. Environmental identity was an important factor for all women, as well as men from China and Brazil. However, its association with sustainable diet intentions was small. Finally, environmental concern was only found to influence sustainable diet intentions of British men. Overall, as each group was influenced by a unique set of factors, this analysis suggests that gender differences in the factors that influence sustainable diet intentions are not consistent across countries.

**Table 4:** Total effects of subjective norms, diet-related identity, perceived status of meat consumption, environmental identity, and environmental concern on sustainable diet intentions for men and women in the UK, China, Sweden and Brazil.

	Men			Women		
	B (SE)	ß	95% CI	B (SE)	ß	95% CI
Subjective norms**→** Sustainable diet intentions						
UK	−0.111 (0.139)	−0.121	−0.330, 0.168	−0.101 (0.067)	−0.095	−0.212, 0.045
China	−0.085 (0.360)	−0.027	−0.528, 0.284	0.209 (0.407)	0.140	−0.228, 0.528
Sweden	0.686 (0.675)	0.419*	0.093, 2.692	−0.154 (0.279)	−0.086	−0.393, 0.210
Brazil	0.138 (0.252)	0.094	−0.152, 0.625	−0.228 (0.101)	−0.222*	−0.469, −0.056
Diet-related identity**→**Sustainable diet intentions						
UK	−0.686 (0.052)	−0.837*	−0.924, −0.757	−0.607 (0.036)	−0.811*	−0.873, −0.743
China	−1.195 (0.318)	−0.873*	−1.339, −0.612	−0.871 (0.250)	−0.716*	−1.031, −0.463
Sweden	−1.068 (0.321)	−0.989*	−2.029, −0.784	−0.761 (0.068)	−0.854*	−0.955, −0.759
Brazil	−1.095 (0.240)	−0.810*	−1.090, −0.627	−0.722 (0.078)	−0.734*	−0.840, −0.631
Perceived status of meat consumption**→** Sustainable diet intentions						
UK	0.354 (0.192)	0.272*	0.027, 0.572	0.301 (0.080)	0.247*	0.129, 0.382
China	0.978 (0.152)	0.745*	0.550, 0.957	1.125 (0.322)	0.629*	0.421, 0.920
Sweden	1.419 (1.244)	0.663*	0.277, 3.385	−0.025 (0.386)	−0.011	−0.320, 0.278
Brazil	0.749 (0.410)	0.461*	0.137, 1.029	−0.244 (0.170)	−0.144	−0.403, 0.034
Environmental identity**→** Sustainable diet intentions						
UK	−0.024 (0.046)	−0.023	−0.123, 0.068	0.100 (0.042)	0.097*	0.022, 0.182
China	0.325 (0.141)	0.163*	0.040, 0.317	0.444 (0.191)	0.186*	0.047, 0.383
Sweden	0.026 (0.146)	0.036	−0.308, 0.199	0.164 (0.074)	0.119*	0.011, 0.216
Brazil	0.223 (0.115)	0.180*	0.031, 0.339	0.271 (0.070)	0.221*	0.127, 0.336
Environmental concern**→** Sustainable diet intentions						
UK	0.137 (0.044)	0.143*	0.052, 0.229	0.071 (0.045)	0.065	−0.021,0.138
China	0.016 (0.116)	0.009	−0.126, 0.120	0.063 (0.112)	0.033	−0.091, 0.160
Sweden	0.038 (0.154)	0.033	−0.183, 0.237	0.018 (0.068)	0.015	−0.095, 0.116
Brazil	−0.180 (0.108)	−0.104	−0.235, 0.002	−0.068 (0.110)	−0.031	−0.133, 0.053

*p < 0.05. Results based off analyses with 1,000 bootstrap samples.

## 4 Discussion

The aim of this study was to explore how intentions to follow a sustainable diet vary across gender and countries. Due to the results of the CFA, intentions to follow a sustainable diet were measured as intentions to follow a vegetarian or vegan diet. It examined gender and country differences in the influence of subjective norms, diet-related identity, perceived status of meat consumption, environmental identity and environmental concern, and whether gender differences were consistent across the UK, China, Sweden and Brazil. Some support was found for gender differences in the factors that influence diet intentions, although the differences were generally small. Support for cross-country differences was stronger, as the importance of several factors that influence a person’s sustainable diet intentions were different in the UK, China, Sweden and Brazil. Furthermore, gender differences in the influence of the different factors varied across the four countries.

Overall, diet-related identity showed the strongest influence on sustainable diet intentions in all groups, across both gender and culture. The strong influence of this variable supports previous research that found that behavior-specific identity is one of the strongest predictors of engagement with pro-environmental behavior (Whitmarsh and O’Neill, 2010). This suggests that viewing meat-eating as part of your identity makes people resistant to forming any intentions to reduce meat intake. Perceived status of meat consumption had the second strongest influence in all groups, although the direction of this relationship was unexpected. The results showed that those who viewed meat-eating as higher status tended to express stronger intentions to follow a vegetarian or vegan diet. One possible explanation of this may be that meat eaters do not tend to consider the status of meat as it is perceived to be a normal and necessary part of diet (Piazza et al., 2015). It may instead only be those who are considering a more sustainable diet that are made aware of the lower status and increased stigma associated with a diet without meat (Markowski and Roxburgh, 2019). One further notable finding of this study was the generally low or non-significant influence of subjective norms, contradicting the TPB (Ajzen, 1991) and its well-established relationship with behavior intentions in other behavioral domains (Manning, 2009; Masud et al., 2016). While the role of norms in shaping diet behavior has previously been contested for a lack of quality research (Robinson, 2015), a recent systematic review found that many studies that adopt the TPB to explain sustainable diet intentions provide evidence for the influence of subjective norms (Biasini et al., 2021). One possible explanation for the inconsistency is that social norms are particularly influential in collectivist cultures (Farrukh et al., 2019) or set in a collectivist context (McAuliffe et al., 2003), meaning that more individualistic situations or behaviors might be less influenced by social norms or people may be less willing to admit to the influence of norms. Supporting this notion is that when directly asked what people think influences their dietary choices, social norms are disregarded, which does not take away from possible influences of norms, despite people’s belief to the contrary (Croker et al., 2009). More research is needed to shed light on the role of social norms in relation to sustainable dietary choices.

Gender differences were only found in the influence of diet-related identity and perceived status of meat consumption. In both cases, these variables were stronger predictors of sustainable diet intentions in men than in women. In line with previous research, the results suggest that a diet-related identity involving meat is more important to men than it is to women, supporting the theory that meat has an important association to a masculine identity (Rozin et al., 2012). However, this gender difference was much smaller than expected, with a large overlap in the confidence intervals of the parameters, given the well-established association between meat and masculinity both in theory and in previous research. The gender difference in the influence of perceived status of meat consumption is more difficult to interpret, not least because of the unexpected direction of the association. As theorized, meat consumption may be so normalized that status is only considered by those intending to reduce their intake. This may have a stronger effect in men if the status of meat consumption is more strongly linked to their identity. This is however speculation and further research is needed to explore this factor in more detail. Overall, the lack of major gender differences in the factors that influence diet intentions suggests that gender plays a less important role in this behavioral domain than in other pro-environmental behaviors (Zelezny et al., 2000; Bloodhart and Swim, 2020; Modlinska et al., 2020). This could be due to rising popularity of plant-based diets among both genders (Jones, 2020), which could have weakened the meat-gender link.

Differences in the factors influencing sustainable diet intentions were larger across countries than between genders. The influence of diet-related identity, perceived status of meat consumption, environmental identity and environmental concern all differed across the four countries. While diet-related identity remained the most influential factor in all countries, the extent of this influence varied with the strongest effect in Sweden and the weakest effect in Brazil. This result was surprising as Brazil has a food culture of high beef consumption (Travassos et al., 2020), suggesting that meat-eating would be an important and influential part of identity in this country. However, the overall differences between the four countries were small. Cross-country differences were larger for the influence of perceived status of meat consumption on sustainable diet intentions. This factor was particularly important in China and to a lesser extent in the UK and Sweden. The factor was not significantly associated with sustainable diet intentions in Brazil. The observed differences may be a product of the food cultures within the four countries that places different levels of value on meat. The importance of general environmental identity and environmental concern for sustainable diet intentions was small yet varied, with significant effects in three and two countries, respectively. This small effect suggests that, overall, sustainable diet intentions are not strongly driven by environmental considerations, contrasting previous research which shows that environmental values are a key dimension in environmental engagement (Milfont, 2012). Notably, environmental identity had the strongest influence in China and Brazil, the countries with the lowest GDP *per capita* out of the four (International Monetary Fund, 2022), despite the suggestion that environmental factors may be more influential in affluent countries as countries are able to focus on environmental issues once basic needs are met (Inglehart, 1995; Diekmann and Franzen, 1999). One explanation for this result could be that this theory applies only at the country scale, and thus within countries that have a lower focus on environmental issues, an individual’s environmental identity plays a more important role on whether they will engage in environmental behaviors. The lack of effect of subjective norms in any country contradicts both previous findings that this influence depends on the affluence of the country (Liobikiene et al., 2016) and the theory that food cultures would result in different norms about meat-eating.

Overall, the most valuable finding of this study is that gender differences do not seem to be consistent across countries. Despite the overall analysis showing that diet-related identity has a stronger influence on sustainable diet intentions in men than in women, this gender difference was only significant in Brazil. This suggests that the association between meat-eating and masculine identity and its influence on behavior may not be as universal as previously suggested (Rozin et al., 2012; Modlinska et al., 2020). Likewise, gender differences in the influence of perceived status of meat consumption also varied across the four countries, with this variable showing an equal effect for the two genders in the UK and China but only influencing men’s intentions in Sweden and Brazil. While no gender differences in the influence of environmental identity appeared in the overall analysis, there was a significant, albeit small, gender difference in the UK with this variable only significantly influencing women. This finding provides some support for the theory that women may have internalized a greater care for the environment as part of a female identity associated with caretaking (Bloodhart and Swim, 2020). However, this effect is not universal suggesting that the association between female and environmental identity varies between cultural contexts. The influence of subjective norms was generally small, as shown by the overall analysis, but gender differences were found in Sweden and Brazil with subjective norms only significantly influencing Swedish men and Brazilian women. Notably, the direction of this relationship in Brazilian women contradicted the proposed relationship of subjective norms in the TPB (Ajzen, 1991), with higher subjective norms in favor of sustainable diets resulting in lower intentions to adopt this diet, suggesting that this group may be reactive to feelings of social pressure. However, previous research from Brazil has not identified this relationship, instead finding that higher subjective norms predict intentions to engage in other pro-environmental behaviors (da Silva Tamashiro et al., 2013; De Farias et al., 2019) questioning the reliability of this finding. One notable finding from this study is that no gender differences were found within China. This appears to be in line with previous research showing that typical gender patterns in Western countries do not necessarily apply to China. For example, unlike in Western countries, men tend to exhibit higher environmental concern and knowledge than women (Xiao and Hong, 2010; Shields and Zeng, 2012). These findings demonstrate a different relationship between gender and pro-environmentalism in China which may explain the lack of gender difference in the more environmentally driven variables. However, the lack of gender difference in other variables may instead suggest that food attitudes and conventions may be more universal across genders within China.

### 4.1 Limitations and future directions

This study provides valuable insights into the variables that influence the intention to choose a sustainable diet, through following a vegan or vegetarian diet. However, it is important to distinguish between intentions and behaviors given the well-established intention-behavior gap, and acknowledge that overall only around half of intentions translate into future behaviors (Sheeran and Webb, 2016). As a result, we cannot be certain that these same variables contribute to driving intentions into behaviors. However, forming intentions is an important first step in behavior change (Ajzen, 1991), meaning that these results still provide valuable insight into the factors that influence sustainable diet behaviors. Another limitation is the relatively low reliabilities of some of the model factors, particularly perceived status of meat consumption and subjective norms. This may be due to the specific items used to measure these factors and may have resulted in high standard errors for some groups. This brings into question how well these variables measure the intended factors. Future research should aim to include more items in the measurement of these variables to ensure reliability. Furthermore, despite this study aiming to focus on a broad range of sustainable diet behaviors, they could not be combined into a reliable factor. This means we are unable to determine whether the results in this study would generalize beyond adopting a vegetarian or vegan diet to other sustainable diet behaviors. As these behaviors could be considered a radical behavior change, it is important to explore whether the same factors influence other, less radical changes toward a more sustainable diet. This highlights a need for future research to explore the variables that influence intentions to engage in these behaviors and how gender and culture effects this. A further limitation of this study is that the study was conducted in a small number of countries. The four countries of the UK, China, Sweden and Brazil were selected based on their dietary cultures, climate ambitions, and rates of development; and the research has shown that there are differences between these countries. However, future research is needed to determine the specific cultural factors underlying the differences in sustainable diet intentions, including but not limited to affluence, Hofstede’s (1980, 2001) individualism versus collectivism and masculinity versus femininity dimensions, and Inglehart’s postmodern (Inglehart, 1997) and traditional versus secular-rational values (Haerpfer et al., 2020).

Overall, the results from this study indicate that there are both gender and cross-country differences in the variables that influence intentions to adopt more sustainable plant-based diets, with cross-country differences being larger than between genders. Importantly, the study finds that gender differences vary across the four countries of the UK, China, Sweden and Brazil. Understanding how these groups differ in their reasons for forming sustainable diet intentions can hopefully act as an important first step in reducing any gender or cultural gap in the adoption of these behaviors. For example, more targeted interventions could be considered based on the relative importance of the different factors underlying sustainable diet-intentions across the different gender and country groups.

## Data availability statement

The data analyzed in this study is subject to the following licenses/restrictions: all data and accompanying will be made available to the UK Data Service (https://ukdataservice.ac.uk) by the end of the CAST (ES/S012257/1) project, as per the requirement of the ESRC. The data will be deposited before 31 July 2024. Before then, the data files can be requested from the authors of the paper. Requests to access these datasets should be directed to EC, chardek@cardiff.ac.uk.

## Ethics statement

The studies involving humans were approved by Cardiff University, School of Psychology Research Ethics Committee. The studies were conducted in accordance with the local legislation and institutional requirements. The participants provided their written informed consent to participate in this study.

## Author contributions

EC: Conceptualization, Formal analysis, Methodology, Writing – original draft. CB: Conceptualization, Supervision, Writing – review & editing. KS: Data curation, Methodology, Supervision, Writing – review & editing. WP: Data curation, Methodology, Supervision, Writing – review & editing. CD: Data curation, Methodology, Writing – review & editing.

## Footnotes

^1^This study used Wave 1 of the survey data, collected in 2020, instead of Wave 2, collected in 2021, as was intended in the pre-registration, as some items were not available in the second wave.

^2^This result was reported to three decimal places to include one significant figure.

## Supplementary material

Supplementary DATA SHEET 1 Sample demographics, correlations between variables, means and standard deviations of variables across groups, and a two-way ANOVA of these variables across gender and country.
