# Supplementary material for: Gender and cross-country differences in the determinants of sustainable diet intentions: a multigroup analysis of the UK, China, Sweden, and Brazil
Source: Front Psychol. 2024 Feb 29;15:1355969. doi: 10.3389/fpsyg.2024.1355969 (PMC10937452; doi:10.3389/fpsyg.2024.1355969)
Supplement: Supplementary DATA SHEET 1 — Sample demographics, correlations between variables, means and standard deviations of variables across groups, and a two-way ANOVA of these variables across gender and country. [file Data_Sheet_1.docx]

Supplementary Material

**Table A.**

*Sample demographics including age, gender, income and region*

|  | N | Gender | Age | Income |  | Region |  |  |
| --- | --- | --- | --- | --- | --- | --- | --- | --- |
|  |  | % female | M(SD) | % sample |  | % sample |  |  |
| *Total* | | | | | | | | |
|  | 4569 | 50.4 | 46.0 (15.75) |  |  |  |  |  |
| *UK* | | | | | | | | |
|  | 1842 | 52.3 | 51.0 (15.64) | Under £10,000 | 7.1 | East Anglia | 4.9 |  |
|  |  |  |  | £10,000 - £19,999 | 19.7 | East Midlands | 3.9 |  |
|  |  |  |  | £20,000 - £29,999 | 21.4 | London | 6.8 |  |
|  |  |  |  | £30,000 - £39,999 | 16.5 | North East | 2.4 |  |
|  |  |  |  | £40,000 - £49,999 | 10.9 | North West | 6.2 |  |
|  |  |  |  | £50,000 - £59,999 | 7.1 | South East | 7.8 |  |
|  |  |  |  | £60,000 - £79,999 | 5.7 | South West | 5.0 |  |
|  |  |  |  | £80,000 - £99,999 | 3.6 | West Midlands | 5.2 |  |
|  |  |  |  | £100,000 or more | 1.7 | Yorkshire & Humberside | 4.5 |  |
|  |  |  |  | Prefer not to say | 5.4 | Northern Ireland | 1.6 |  |
|  |  |  |  |  |  | Scotland | 25.8 |  |
|  |  |  |  |  |  | Wales | 25.1 |  |
| *China* | | | | | | | | |
|  | 807 | 45 | 39.7 (11.73) | Under RMB 48,000 | 4.3 | Central | 12.3 |  |
|  |  |  |  | RMB 48,000 – RMB 95,999 | 11.2 | East | 33.5 |  |
|  |  |  |  | RMB 96,000 – RMB 107,999 | 6.6 | North | 15.0 |  |
|  |  |  |  | RMB 108,000 – RMB 119,999 | 10.9 | North East | 9.4 |  |
|  |  |  |  | RMB 120,000 – RMB 143,999 | 17.0 | North West | 5.8 |  |
|  |  |  |  | RMB 144,000 – RMB 215,999 | 25.9 | South | 13.4 |  |
|  |  |  |  | RMB 216,000 or more | 22.8 | South West | 9.9 |  |
|  |  |  |  | Prefer not to say | 0.1 |  |  |  |
| *Sweden* | | | | | | | | |
|  | 963 | 49.7 | 48.3 (16.60) | Under 100,000SEK | 5.2 | East | 22.8 |  |
|  |  |  |  | 100,000 – 199,999SEK | 21.2 | Middle | 17.9 |  |
|  |  |  |  | 200,000 – 299,999SEK | 17.7 | North | 11.4 |  |
|  |  |  |  | 300,000 – 399,999SEK | 19.7 | South | 21.2 |  |
|  |  |  |  | 400,000 – 499,999SEK | 10.5 | South East | 7.1 |  |
|  |  |  |  | 500,000SEK or more | 22.7 | West | 18.3 |  |
|  |  |  |  | Prefer not to say | 1.9 |  |  |  |
| *Brazil* | | | | | | | | |
|  | 957 | 52 | 39.3 (13.69) | Under 20,000 BRL | 26.8 | Centro-Oeste | 8.4 |  |
|  |  |  |  | 20,000 – 60,999 BRL | 40.1 | Nordeste | 26.0 |  |
|  |  |  |  | 61,000 – 99,999 BRL | 13.1 | Norte | 4.9 |  |
|  |  |  |  | 100,000 – 130,999 BRL | 6.2 | Sudeste | 45.6 |  |
|  |  |  |  | 131,000 – 160,999 BRL | 2.5 | Sul | 13.8 |  |
|  |  |  |  | 161,000 – 200,999 BRL | 3.2 |  |  |  |
|  |  |  |  | 201,000 – 230,999 BRL | 0.8 |  |  |  |
|  |  |  |  | 231,000 – 260,999 BRL | 1.0 |  |  |  |
|  |  |  |  | 261,000 BRL or more | 1.4 |  |  |  |
|  |  |  |  | Prefer not to say | 2.7 |  |  |  |

**Table B.**

*Correlations between sustainable diet intentions, subjective norms, diet-related identity, perceived status of meat consumption, environmental identity, and environmental concern.*

|  | 1 | 2 | 3 | 4 | 5 |
| --- | --- | --- | --- | --- | --- |
| 1. Sustainable diet intentions |  |  |  |  |  |
| 2. Subjective  norms | -.01 |  |  |  |  |
| 3. Diet-related identity | -.60* | -.26* |  |  |  |
| 4. Perceived status of meat consumption | .09* | -.43* | .19* |  |  |
| 5. Environmental identity | .35* | -.02 | -.25* | -.06* |  |
| 6. Environmental concern | .27* | .09* | -.25* | -.06* | .50* |

Note: *p<.001.

**Table C.**

*Means and standard deviations of subjective norms, diet-related identity, perceived status of meat consumption, environmental identity, environmental concern and sustainable diet intentions across all sample groups.*

|  |  | Subjective norms | Diet-related identity | Perceived status of meat consumption | Environmental identity | Environmental concern | Sustainable diet intentions |
| --- | --- | --- | --- | --- | --- | --- | --- |
|  |  | M (SD) | M (SD) | M (SD) | M (SD) | M (SD) | M (SD) |
| Gender | | | | | | | |
|  | Men | 3.40 (.02) | 3.40 (.02) | 2.51 (.02) | 3.58 (.02) | 3.31 (.02) | 2.24 (.02) |
|  | Women | 3.57 (.02) | 3.01 (.02) | 2.37 (.02) | 3.73 (.02) | 3.59 (.02) | 2.52 (.03) |
| Country | | | | | | | |
|  | UK | 3.75 (.02) | 3.29 (.03) | 2.30 (.02) | 3.46 (.02) | 3.34 (.02) | 2.00 (.03) |
|  | China | 2.81 (.03) | 3.33 (.03) | 2.38 (.03) | 4.15 (.02) | 3.31 (.03) | 2.66 (.04) |
|  | Sweden | 3.65 (.03) | 2.92 (.04) | 2.63 (.03) | 3.39 (.03) | 3.26 (.03) | 2.70 (.04) |
|  | Brazil | 3.37 (.04) | 3.20 (.03) | 2.55 (.03) | 3.89 (.03) | 3.99 (.02) | 2.55 (.04) |
| Group | | | | | | | |
|  | UK Men | 3.69 (.03) | 3.56 (.04) | 2.38 (.03) | 3.37 (.03) | 3.21 (.03) | 1.78 (.03) |
|  | UK Women | 3.81 (.03) | 3.04 (.04) | 2.23 (.03) | 3.54 (.03) | 3.46 (.03) | 2.20 (.04) |
|  | China Men | 2.78 (.04) | 3.40 (.04) | 2.44 (.04) | 4.09 (.03) | 3.19 (.03) | 2.62 (.05) |
|  | China Women | 2.84 (.04) | 3.25 (.05) | 2.31 (.04) | 4.21 (.03) | 3.45 (.04) | 2.71 (.06) |
|  | Sweden Men | 3.52 (.04) | 3.16 (.05) | 2.73 (.04) | 3.23 (.04) | 3.09 (.04) | 2.49 (.06) |
|  | Sweden Women | 3.79 (.04) | 2.69 (.05) | 2.54 (.04) | 3.54 (.04) | 3.42 (.04) | 2.92 (.06) |
|  | Brazil Men | 3.31 (.05) | 3.33 (.05) | 2.59 (.05) | 3.85 (.04) | 3.87 (.04) | 2.46 (.06) |
|  | Brazil Women | 3.42 (.05) | 3.08 (.05) | 2.51 (.05) | 3.92 (.04) | 4.10 (.03) | 2.64 (.05) |

**Table D.**

*Two-way ANOVA results of the difference of subjective norms, diet-related identity, perceived status of meat consumption, environmental identity, environmental concern and sustainable diet intentions across gender and country, and the interaction between gender and country*

|  |  | df | F | p |  |
| --- | --- | --- | --- | --- | --- |
| Subjective norms | | | | | |
|  | Gender | 1 | 21.67 | <.001** |  |
|  | Country | 3 | 187.82 | <.001** |  |
|  | Gender*Country | 3 | 2.05 | .10 |  |
| Diet-related identity | | | | | |
|  | Gender | 1 | 109.52 | <.001** |  |
|  | Country | 3 | 30.00 | <.001** |  |
|  | Gender*Country | 3 | 7.21 | <.001** |  |
| Perceived status of meat consumption | | | | | |
|  | Gender | 1 | 22.53 | <.001** |  |
|  | Country | 3 | 33.57 | <.001** |  |
|  | Gender*Country | 3 | .58 | .63 |  |
| Environmental identity | | | | | |
|  | Gender | 1 | 35.98 | <.001** |  |
|  | Country | 3 | 164.48 | <.001** |  |
|  | Gender*Country | 3 | 3.28 | .02* |  |
| Environmental concern | | | | | |
|  | Gender | 1 | 101.00 | <.001** |  |
|  | Country | 3 | 159.24 | <.001** |  |
|  | Gender*Country | 3 | .73 | .54 |  |
| Sustainable diet intentions | | | | | |
|  | Gender | 1 | 60.73 | <.001** |  |
|  | Country | 3 | 118.97 | <.001** |  |
|  | Gender*Country | 3 | 5.32 | .001** |  |

Note: *p<.05, **p<.001
